# Meta-QTLs and candidate genes for stripe rust resistance in wheat

**DOI:** 10.1038/s41598-021-02049-w

**Published:** 2021-11-25

**Authors:** Irfat Jan, Gautam Saripalli, Kuldeep Kumar, Anuj Kumar, Rakhi Singh, Ritu Batra, Pradeep Kumar Sharma, Harindra Singh Balyan, Pushpendra Kumar Gupta

**Affiliations:** Department of Genetics and Plant Breeding, Ch. Charan Singh University, Meerut, 250004 India

**Keywords:** Genetics, Plant sciences

## Abstract

In bread wheat, meta-QTL analysis was conducted using 353 QTLs that were available from earlier studies. When projected onto a dense consensus map comprising 76,753 markers, only 184 QTLs with the required information, could be utilized leading to identification of 61 MQTLs spread over 18 of the 21 chromosomes (barring 5D, 6D and 7D). The range for mean R^2^ (PVE %) was 1.9% to 48.1%, and that of CI was 0.02 to 11.47 cM; these CIs also carried 37 Yr genes. Using these MQTLs, 385 candidate genes (CGs) were also identified. Out of these CGs, 241 encoded known R proteins and 120 showed differential expression due to stripe rust infection at the seedling stage; the remaining 24 CGs were common in the sense that they encoded R proteins as well as showed differential expression. The proteins encoded by CGs carried the following widely known domains: NBS-LRR domain, WRKY domains, ankyrin repeat domains, sugar transport domains, etc. Thirteen breeders’ MQTLs (PVE > 20%) including four pairs of closely linked MQTLs are recommended for use in wheat molecular breeding, for future studies to understand the molecular mechanism of stripe rust resistance and for gene cloning.

## Introduction

Wheat is grown on > 200 mha of land globally and provides 20% of the daily protein and calories for the growing world population^1,2^. In production, wheat is the second most important crop after maize (FAO-2021, http://www.fao.org/3/cb3672en/cb3672en.pdf). Three rust diseases (leaf rust, stem rust and stripe rust) represent a major biotic stress affecting wheat yield worldwide. Among the three rusts, stripe rust (also known as yellow rust) caused by *Puccinia striiformis* f. sp. tritici (Pst) is the most devastating and widely occurring disease in major wheat growing regions of the world. The yield losses due to stripe rust can be upto 70% under severe epidemic conditions, adversely affecting grain-filling duration, leading to poor grain yield and quality^3^. Under severe conditions, the yield losses may approach 100%, when the infection occurs at the seedling stage and environmental conditions are conducive for the pathogen to persist until maturity^4,5^.

At least 140 stripe rust pathotypes are known globally; of these, ~ 28 pathotypes occur in India alone^5–7^. Transcaucasia including the border of Eastern Europe and Western Asia was initially suggested to be the centre of origin of this pathogen^8,9^. However, later, Himalayan region was confirmed to be the actual centre of origin^10^. Even some of the aggressive stripe rust races (e.g., Warrior and Kranich) which were recently identified in Europe are known to have originated in the Himalayan region^11–13^. This suggested that *Pst* can migrate long distances leading to its current word-wide occurrence^14^.

Stripe rust occurs in regions, where cool and humid conditions persist during crop season. In India, it is a major disease in the North West Plain Zones (NWPZ) including Punjab, Haryana, and Western Uttar Pradesh, which are the major wheat growing areas. A major outbreak of this disease in India was witnessed in NWPZ during 2006 and in the Northern Hills Zone (NHZ) during 2012–2013^5,15,16^.

Eighty three Yr genes for resistance against stripe rust in wheat have already been identified globally, which are distributed on all the 21 wheat chromosomes^17^. More than 15 of these Yr genes have been derived from alien species^18^. Nine Yr genes (*Yr5/Yrsp, Yr7, Yr10, Yr15, Yr36, Yr18, YrU1* and *Yr46*) have also been cloned^19–23^. These cloned Yr genes encode a variety of proteins including those with the following domains: nucleotide binding site leucine rich repeats (NBS-LRR on NLR) domain, kinase like domains, ankyrin repeats*,* WRKY domain and lipid binding domain. Yr genes encoding putative ABC transporter and hexose transporter are also known. A number of other defense genes have also been identified, which are expressed during wheat-Pst interaction^5,23–25^. A network pathway operating during the wheat-Pst interaction was also presented in one of the earlier studies^26^.

Stripe rust resistance has also been treated as a quantitative trait (QT), so that interval mapping has been used to identify > 350 QTLs^27,28^. Several LD-based genome wide association studies (GWAS) for stripe rust resistance have also been conducted, leading to identification of a large number of marker trait associations (MTAs). The activity involving quantitative genetics studies for this trait increased in recent years, with at least five studies reported in 2021^3,29–32^.

Meta-QTL (MQTL) analysis involving known QTLs for resistance against stripe rust in wheat has never been conducted, although it has been conducted for a number of other traits. These other traits earlier used for MQTL analysis included the following: tolerance to abiotic stresses such as drought and heat^33,34^, resistance to a number of diseases including leaf rust^35,36^, tan spot^37^, Fusarium head blight^38^ and powdery mildew^39^. The objective of the present study was to conduct MQTL analysis for resistance against stripe rust in wheat, followed by identification of candidate genes (CGs) for the MQTLs thus identified. A more specific objective was to select a few breeders’ MQTLs and CGs to serve as an important resource to supplement wheat breeding through marker assisted selection (MAS) and for future basic studies to understand the mechanism of stripe rust resistance in bread wheat.

## Results

### QTLs and their distribution on wheat chromosomes

Stripe rust resistance can be either all stage resistance (ASR), which is also described as seedling resistance (SR), or adult plant resistance (APR), which also includes the so-called high temperature adult plant (HTAP) resistance. As a result, in the published literature, the following four different terms have been used for QTLs for stripe rust resistance: SR, ASR, APR and HTAP. However, for the purpose of the present study, we will consider only two classes, ASR (including SR) and APR (including HTAP).

**Figure 1:**
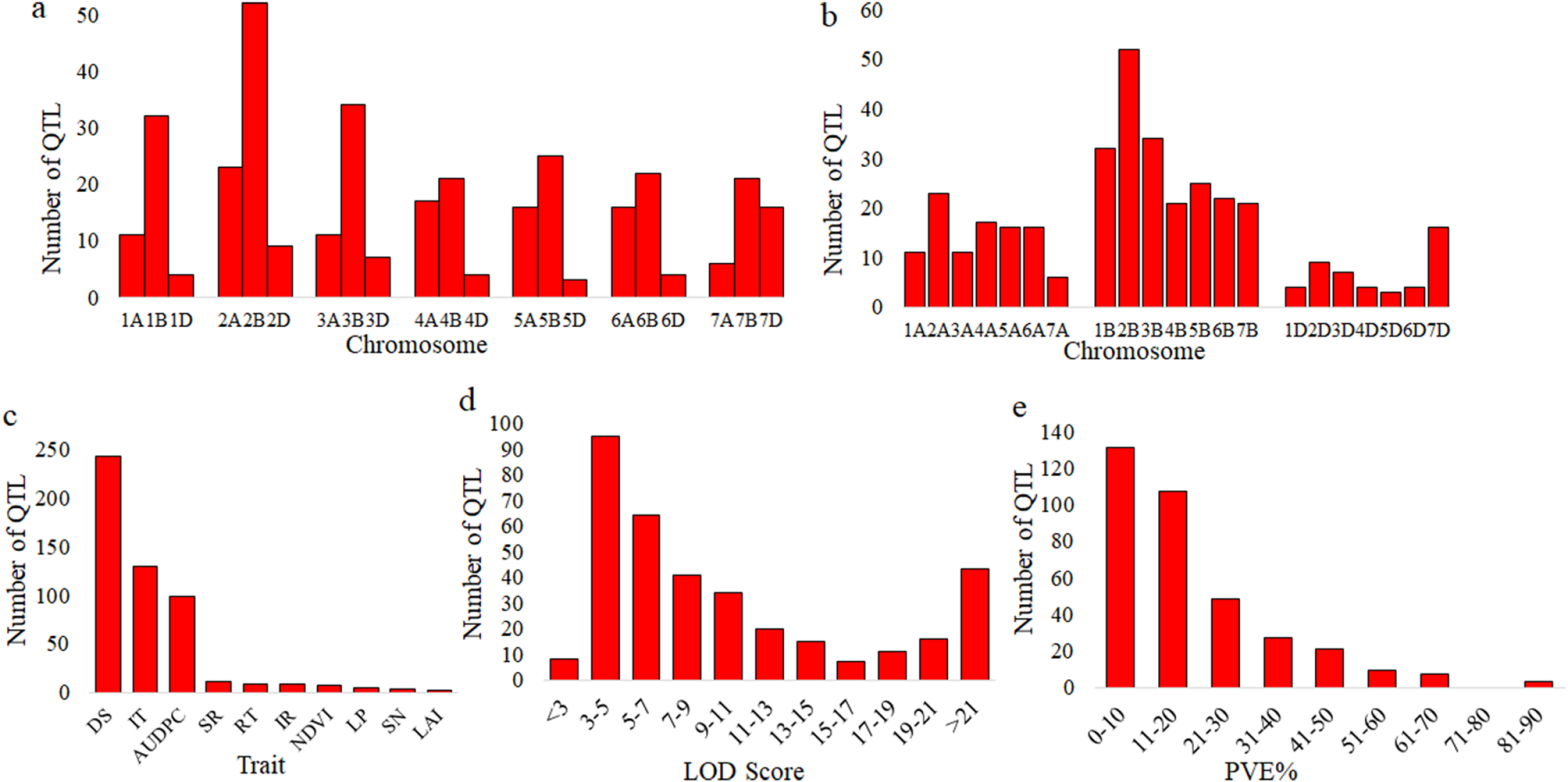
QTLs for stripe rust resistance in wheat. Distribution of QTLs (**a**) on chromosomes of seven homoeologous groups, (**b**) on chromosomes of three sub-genomes, (**c**) for 10 different traits, (**d**) according to logarithm of odds (LOD) values, and (**e**) according to their phenotypic variation explained (PVE%). *DS* disease severity, *IT* infection type, *AUDPC* area under disease progress curve, *SR* stripe rust response, *RT* reaction type, *IR* infection response, *NDVI* normalized difference vegetation index, *LP* latency period, *SN* stripe number per 10 cm^2^ leaf area, *LAI* leaf area infected.

In the present study, using WheatQTLdb and the published literature, a total of 86 studies were available; only 75 studies (73 studies on common wheat + two studies on durum wheat) were found to contain all the required information related to QTLs and were used for the MQTL analysis (Table 1; for details see Supplementary Table S1). The mapping populations in these studies consisted of RILs, DH or F_3_ populations (Table1; Supplementary Table S1). A total of 353 QTLs were available in these 75 studies. The information on the flanking markers, phenotypic variation explained (PVE%) for each QTL along with their confidence intervals (CIs) are presented in Supplementary Table S1. Following are some details about these 353 QTLs: (i) These QTLs were distributed on all the 21 wheat chromosomes, with the total number of QTLs per chromosome ranging from 3 on chromosome 5D to 52 on chromosome 2B (Fig. 1a,b). (ii) The distribution of QTLs among three sub-genomes also widely differed, with 100 (28.3%) QTLs on A sub-genome, 206 QTLs (58.4%) on B sub-genome and 47 QTLs (13.3%) on D sub-genome (Fig. 1b). (iii) The number of QTLs for ten individual traits relevant to stripe rust ranged from 2 QTLs for leaf area infected (LAI) to 243 for disease severity (DS) (Fig. 1c). (iv) LOD score for individual QTLs ranged from 2 to 62 with 47% of QTLs showing a LOD score of 2–7 (Fig. 1d). (v) The proportion of phenotypic variance explained (PVE%) by individual QTLs ranged from 1 to 88% (average = 6%) and followed the characteristic L-shaped distribution, with most (67%) QTLs showing a PVE < 30% and only a small fraction representing major genes (PVE > 30%) (Fig. 1e). (vi) A set of 302 QTLs (including 37 QTLs for HTAP resistance) were available for APR and only 25 QTLs were available for ASR (including 8 QTLs described as QTLs for SR). The information on the type of resistance for the remaining 26 QTLs was not available.

**Table 1:** A summary of QTL studies used for MQTL analysis (Details are provided in Supplementary Table S1).

No. of mapping populations (range of population size; no. of studies*)	Parameters	No. of pathotypes used	Methods of QTL analysis
**I. RIL populations**			
59 (92–288; 56)	AUDPC, IT, DS, SR, NDVI, LP, RT	58	ICIM, CIM, SMA, LOCO-LMM, MIM
**II. DH populations**			
18 (78–1020; 14)	IT, DS, SR, IR, AUDPC, RT, NDVI, LAI	22	ICIM, CIM
**III. F**_**3**_ **populations**			
7 (136–326; 7)	IT, AUDPC, DS, SN	18	ICIM, SIM, CIM

*RIL* recombinant inbred line, *DH* doubled haploid, *AUDPC* area under disease progress curve, *IT* infection type, *DS* disease severity, *SR* stripe rust response, *NDVI* normalized difference vegetation index, *LP* latency period, *RT* reaction type, *IR* infection response, *LAI* leaf area infected, *SN* number of stripes per 10 cm^2^ leaf area, *ICIM* inclusive composite interval mapping, *CIM* composite interval mapping, *SMA* single marker analysis, *LOCO-LMM* leave one chromosome out-linear mixed model, *MIM* multiple interval mapping, *SIM* simple interval mapping.

*In one of the studies, two different populations, i.e. RIL and DH, both are used. Therefore, this study is counted two times, i.e., in I and II.

### Consensus map and projection of QTLs on consensus map

**Figure 2:**
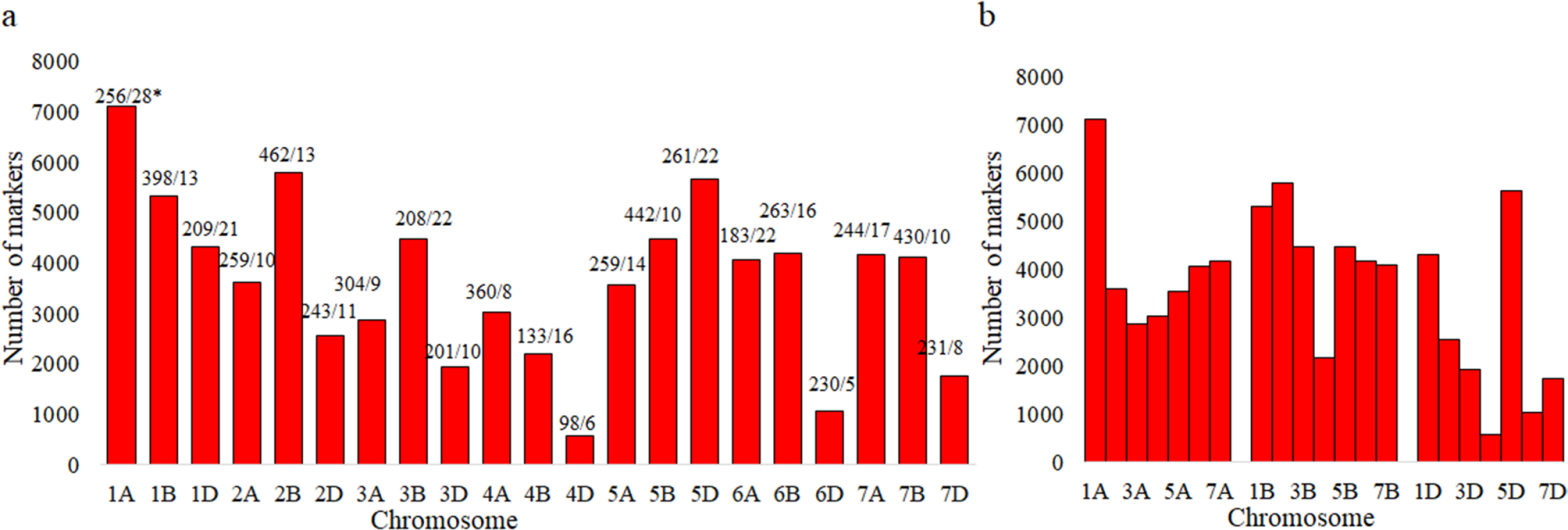
Summary of the number of markers on each of the 21 wheat chromosome (**a**) grouped according to homoeologous chromosomes, and (**b**) grouped according to the three sub-genomes. * = Chr. Length/marker density.

The dense consensus map had 76,753 markers, including 3,526 DArT, 65,459 SNP, 3,975 SSR, and the remaining 3,793 markers representing a group including AFLP, STS, TRAP, etc., which have been used only rarely for QTL interval mapping. The total length of the consensus map was 5774 cM; the size of the 21 individual linkage groups ranged from 98 cM (4D) to 462 cM (2B) (Fig. 2a). The marker densities ranged from 5 markers per cM for 6D to 28 markers per cM for 1A (Fig. 2a). Average marker densities for three sub-genomes also differed, with lowest marker density for D sub- genome (11.85 markers per cM) followed by B sub-genome (14.28 markers per cM) and A sub-genome (15.42 markers per cM) (Fig. 2b). Only 214 QTLs from a total of 353 QTLs could be projected onto the consensus map and were used for MQTL analysis.

### MQTLs and their distribution on wheat genome

**Figure 3:**
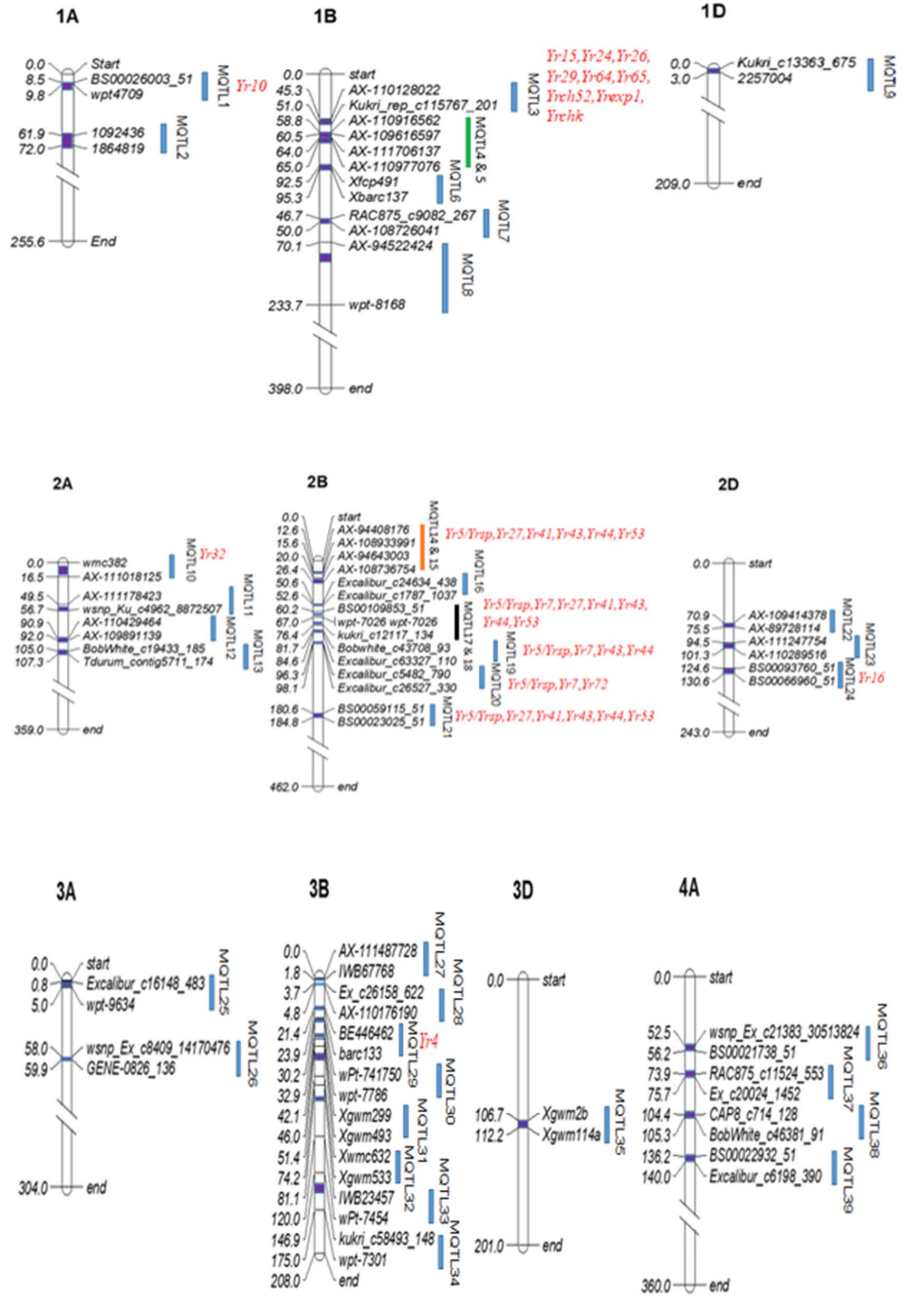

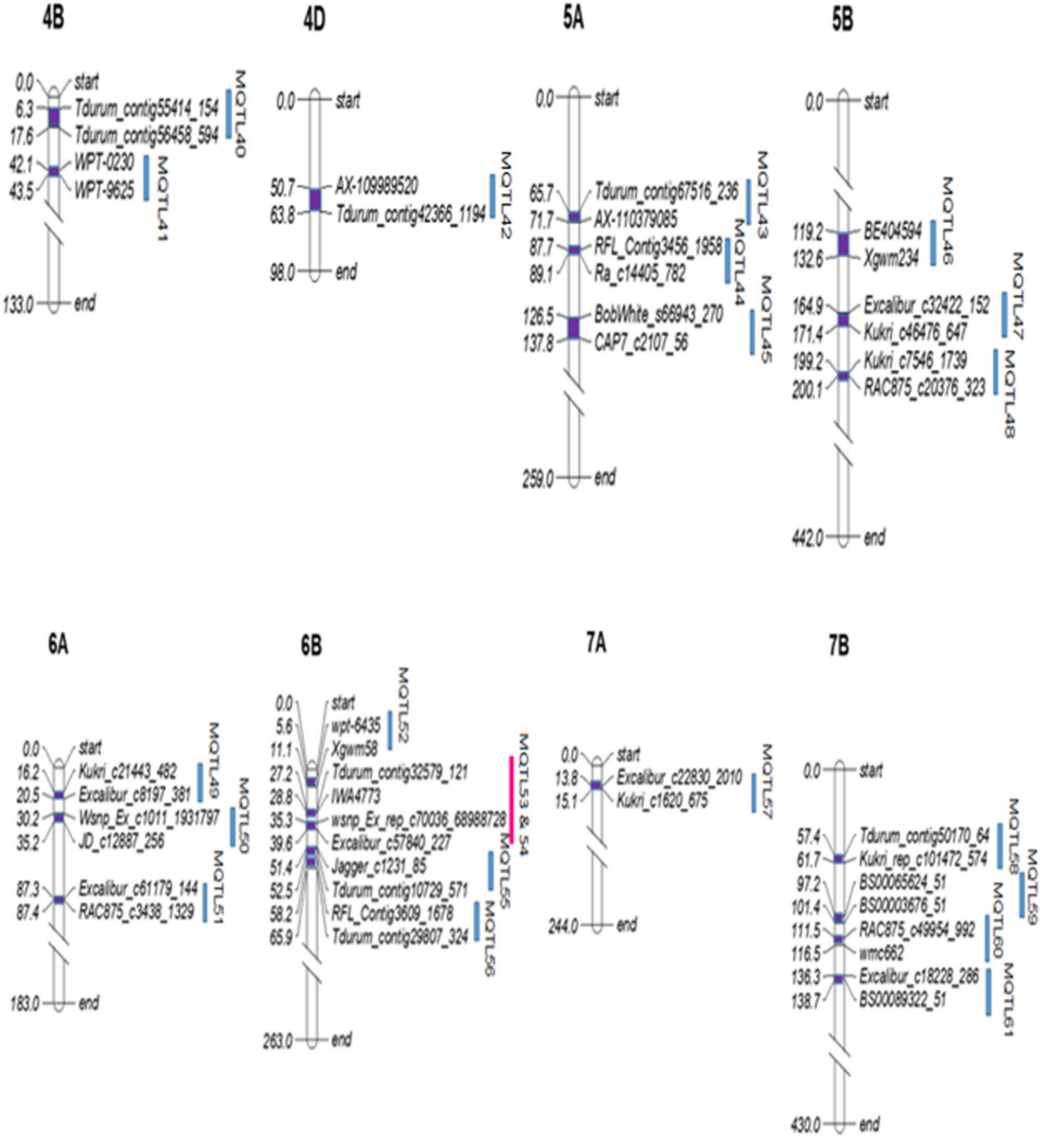
Distribution of 61 MQTLs on 18 chromosomes of wheat. The dark blue rectangular blocks inside each chromosome indicate MQTL regions and the light blue vertical bars on the right of each chromosomes indicate marker intervals. Marker intervals, occupying closely linked pairs of QTL are shown by the green, orange, black and pink colors. Only the flanking markers most closely associated with MQTLs have been shown in the figure. The Yr genes located in the marker interval of the MQTLs are indicated in red font.

Out of the 214 QTLs projected on the consensus map, 30 QTLs remained singletons and were therefore removed from further analysis. The remaining 184 QTLs gave 61 MQTLs; these MQTLs were located on 18 of the 21 chromosomes leaving out 5D, 6D and 7D. The number of MQTL on individual chromosomes ranged from 1 to 8 MQTLs and that on three sub-genomes ranged from 6 on D sub-genome to 36 on the B sub-genomes, with A sub-genome carrying 19 MQTLs (Fig. 3; Table 2). The number of QTLs used for individual MQTLs ranged from 2 to 10 (Table 2). Mean R^2^ (PVE %) for individual MQTLs ranged from 1.9% to 48.1% with their CI ranging from 0.02 cM (MQTL51) to 11.47 cM (MQTL40). The 61 MQTLs largely belonged to APR (five of them apparently pleiotropic) with a solitary exception of a QTL, which belonged to ASR. The detailed information for individual MQTLs including their positions on genetic/physical maps is provided in Supplementary Table S2. Eight MQTLs each belonged to four pairs, each pair with two adjacent overlapping MQTLs.

**Table 2:** A summary of the results of MQTL analysis for stripe rust resistance in wheat.

MQTL-chromosome	Interval (cM/Mb)	Peak pos. (cM)/mean R^2^	QTL studies; QTLs represented by MQTL (no. of pathotypes)	Traits associated with the MQTL
MQTL1-1A	8.90–10.10/6.34–584.36	9.48/9.6	4; 4 (3)	DS,IT
MQTL2-1A	62–71.80/483.52–510.46	66.84/8.2	2; 2 (3)	DS
MQTL3-1B	45.31–48.99/660.56–687.79	47.15/27.6	2; 2 (7)	DS,IT
MQTL4-1B	58.91–60.37/394.03–394.33	59.64/15.15	3; 3 (5)	IT,SR,IR,DS,NDVI
MQTL5-1B	64.41–64.71/398.15–571.19	64.56/23.94	6; 6 (12)	IT,DS,AUDPC,NDVI
MQTL6-1B	94.73–95.23/189.34–678.74	94.98/27.35	3; 3 (7)	DS,IT
MQTL7-1B	148.29–148.93/667.21–667.60	148.61/9.8	3; 3 (9)	DS
MQTL8-1B	186.28–189/439.01–670.78	187.64/34.2	2; 2 (4)	DS,IT
MQTL9-1D	0.08–2.93/7.84–423.26	1.5/5.8	2; 2 (3)	DS
MQTL10-2A	5.72–13.22/2.50–36.85	9.47/45	5; 5 (16)	DS,IT
MQTL11-2A	54.38–56.40/18.29–27.44	55.39/14.15	5; 5 (8)	AUDPC,DS
MQTL12-2A	91.70–92.20/695.18–715.29	91.95/13.3	3; 3 (7)	RT,DS
MQTL13-2A	105.29–106.87/30.87–32.11	106.08/41	2; 2 (5)	DS,AUDPC,IT
MQTL14-2B	14.02–14.92/598.57–680.77	14.47/17.9	2; 2 (3)	DS
MQTL15-2B	20.49–25.64/562.26–673.71	23.06/11.25	3; 3 (4)	DS
MQTL16-2B	50.79–52/0.48–16.83	51.4/45.8	2; 2 (3)	DS,IT
MQTL17-2B	64.70–65.58/42.28–771.17	65.14/14.46	4; 5 (6)	DS
MQTL18-2B	74.16–75.98/764.90–771.17	75.07/29.70	10; 10 (23)	DS,AUDPC,IT,SR,IR
MQTL19-2B	81.76–84.56/554.45–763.84	83.16/21.6	4; 4 (8)	DS,AUDPC,IT
MQTL20-2B	96.40–98.14/687.47–777.14	97.27/40.26	5; 5 (15)	DS,IT,NDVI
MQTL21-2B	180.74–183.64/29.04–672.64	182.19/16.8	2; 2 (3)	DS,IT
MQTL22-2D	71.28–72.56/20.76–26.34	71.92/10.2	2; 2 (3)	AUDPC,DS
MQTL23-2D	96.53–100.20/45.88–57.26	98.37/48.1	2; 2 (2)	LP,IT,DS
MQTL24-2D	124.65–130.6/27.92–132.52	127.62/47.2	3; 3 (6)	AUDPC,DS,IT
MQTL25-3A	1.20–2.76/10.14–11.38	1.98/8.05	3; 3 (2)	LP,IT,DS
MQTL26-3A	58.46–59.39/24.61–32.15	58.92/32.6	3; 3 (5)	DS,IT
MQTL27-3B	0.12–0.70/5.59–6.75	0.41/24.5	2; 2 (6)	DS,IT
MQTL28-3B	3.69–4.67/710.98–773.05	4.18/35.7	3; 3 (2)	DS,IT
MQTL29-3B	21.40–23.70/2.89–7.61	22.55/11.1	3; 3 (4)	SR,IR,DS,RT
MQTL30-3B	32.05–32.94/15.05–130.42	32.49/15.98	6; 6 (12)	DS,AUDPC,IT
MQTL31-3B	42.55–43.64/804.80–811.89	43.09/6.825	3; 3 (9)	IT,DS,NDVI
MQTL32-3B	56.95–59.82/77.49–822.58	58.38/8	4; 4 (10)	DS,AUDPC,IT
MQTL33-3B	90.92–91.48/5.59–739.14	91.2/13.675	4; 5 (9)	DS,AUDPC,IT
MQTL34-3B	152.56–156.68/778.29–814.63	154.62/35	2; 2 (4)	DS
MQTL35-3D	107.50–111.81/47.78–603.46	109.65/11.7	2; 2 (8)	DS,AUDPC,IT
MQTL36-4A	52.94–56.52/591.70–596.31	54.73/11.5	2; 3 (1)	SR,IR,DS
MQTL37-4A	74.39–75.54/615.94–617.99	74.97/7	2; 2 (3)	DS,IT
MQTL38-4A	104.42–105.41/667.35–673.97	104.91/3.5	2; 2 (2)	DS,IT
MQTL39-4A	136.30–140.09/719.18–731.70	138.19/12.85	3; 3 (2)	DS,IT
MQTL40-4B	6.11–17.61/23.46–646.14	11.86/48.5	3; 3 (3)	DS,AUDPC,IT
MQTL41-4B	42.45–43.32/149.04–626.78	42.88/14.275	5; 5 (7)	DS,IT
MQTL42-4D	52.82–63.82/456.36–488.50	58.32/22	2; 2 (10)	DS,AUDPC,IT
MQTL43-5A	65.73–71.43/445.28–458.20	68.58/6.4	2; 2 (3)	AUDPC,DS
MQTL44-5A	88.10–88.97/57.92–595.90	88.53/6.4	2; 2 (10)	SR,AUDPC,DS
MQTL45-5A	126.69–137.69/499.45–698.64	132.19/3.9	2; 2 (NA)	DS,AUDPC,IT
MQTL46-5B	126.99–130.34/8.19–12.33	128.66/10.3	4; 4 (9)	AUDPC,DS
MQTL47-5B	164.88–171.45/44.47–479.39	168.16/13.5	3; 3 (10)	DS,IT
MQTL48-5B	199.02–199.81/695.66–695.79	199.41/6.7	2; 2 (2)	DS
MQTL49-6A	16.18–20.28/14.35–18.71	18.23/4.6	3; 3 (4)	DS,IT
MQTL50-6A	30.25–34.82/17.90–24.07	32.53/10.2	3; 3 (3)	LP,IT,DS,AUDPC
MQTL51-6A	87.35–87.37/448.22–550.64	87.36/1.98	2; 2 (13)	DS,IT
MQTL52-6B	5.80–10.99/398.97–418.14	8.39/4.53	4; 4 (15)	AUDPC,DS
MQTL53-6B	27.23–28.58/680.08–705.29	27.9/21.33	4; 4 (10)	DS,AUDPC,IT
MQTL54-6B	35.59–39.53/690.96–694.13	37.56/4.99	2; 2 (2)	AUDPC
MQTL55-6B	51.48–52.95/708.02–720.98	51.95/25	2; 2 (1)	DS,IT
MQTL56-6B	58.08–63.99/465.68–508.61	61.03/25.85	3; 3 (6)	DS,AUDPC,IT
MQTL57-7A	13.85–15.33/3.78–4.47	14.59/6.3	1; 2 (NA)	DS
MQTL58-7B	57.60–61.61/41.10–421.07	59.6/13.5	2; 2 (2)	DS,IT
MQTL59-7B	97.51–101.40/704.28–709.00	99.45/8.86	5; 5 (8)	AUDPC,DS
MQTL60-7B	113.52–116.40/365.38–732.65	113.96/5.7	2; 2 (2)	DS
MQTL61-7B	136.61–138.42/716.65–717.00	137.51/11.3	3; 3 (4)	IT,DS

*NA* not available, *AUDPC* area under disease progress curve, *IT* infection type, *DS* disease severity, *SR* stripe rust response, *NDVI* normalized difference vegetation index, *LP* latency period, *RT* reaction type, *IR* infection response.

### Co-located Yr genes with the MQTLs

Thirty-seven (37) known major Yr genes were found to be located within the marker intervals for MQTLs (on the basis of physical positions of MQTLs and the Yr genes) of the following 24 MQTLs; MQTL3, 5, 6, 8, 10, 12, 14, 15, 17, 18, 19, 20, 21, 24, 28, 29, 32, 33, 35, 40, 41, 45, 58 and 60; 8 Yr genes were located within the marker interval of a single MQTL7 on chromosome 2B (Fig. 3). Since the physical intervals of several MQTLs overlapped with each other, sometimes the same Yr gene is associated with more than one MQTLs, particularly on chromosome 2B. Positions of some of the important cloned Yr genes like *Yr36, Yr18* and *Yr46* with respect to positions of MQTLs could not be identified during the present study.

### Identification of CGs, GO terms and in-silico expression analysis

**Figure 4:**
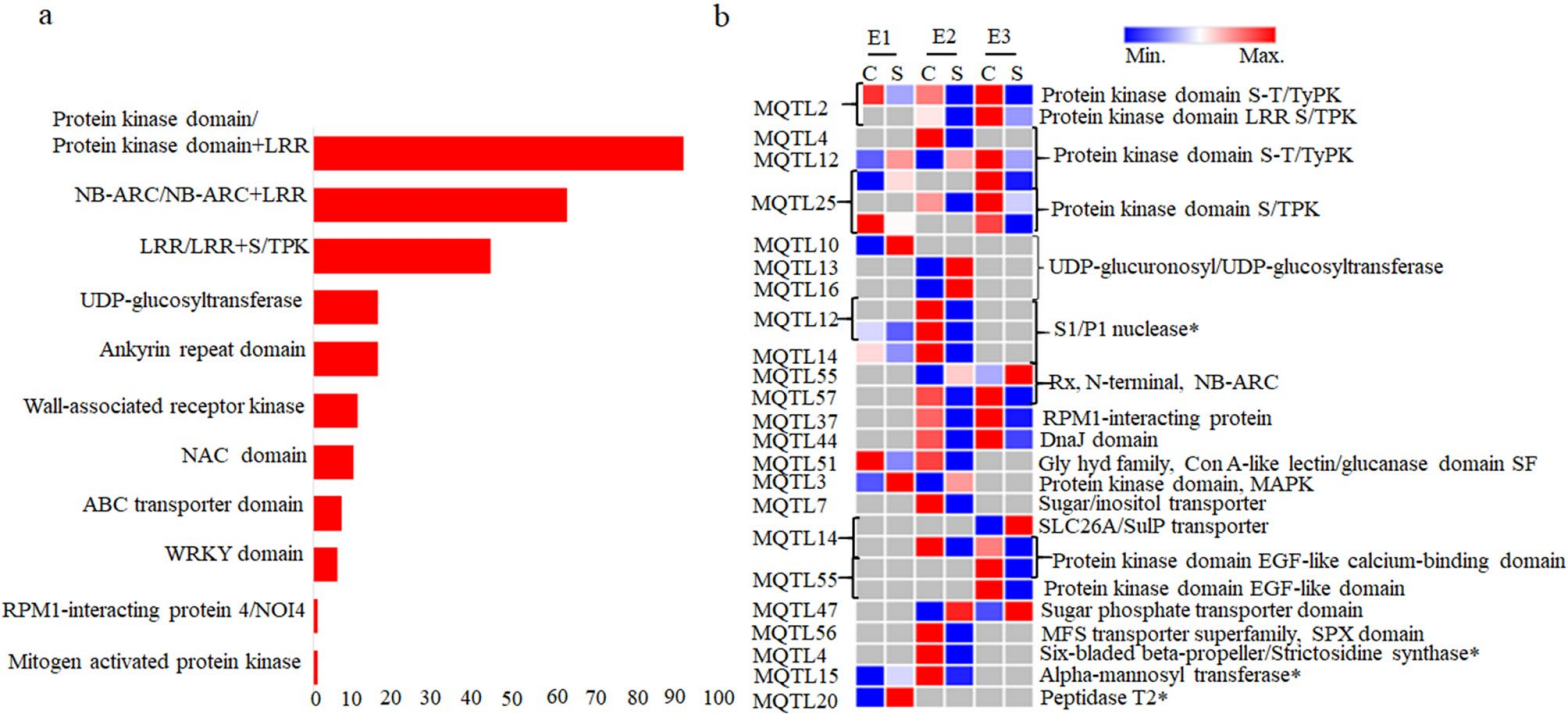
Important candidate genes: (**a**) histogram showing frequencies of CGs encoding proteins with 11 different domains involved in disease resistance; (**b**) heatmap showing 29 important differentially expressed CGs. *C* control, *S* stress, *E1* experiment 1, *E2* experiment 2, *E3* experiment 3, *S-T/TyPK* serine-threonine/tyrosine protein kinase, *S/TPK* serine/threonine protein kinase, *LRR* leucine rich repeats. Genes marked with asterix (*) had FC ≥ 5 or ≤ − 5.

(*i*) *Identification of CGs*. A total of 1581 CGs were available in the genomic regions defined by 60 out of the 61 MQTLs; no CG was available in the solitary remaining MQTL6; 385 of these CGs were selected for a more detailed study. These selected CGs comprised the following two categories: (i) 265 important CGs encoding R proteins (belonging to 48 MQTLs) (Fig. 4a and Supplementary Table S3) and (ii) 144 differentially expressed CGs (DECGs), belonging to 44 MQTLs, based on in silico expression analysis (Supplementary Table S4). Twenty-four (24) CGs were common among the above two categories of genes (Table 3, Supplementary Tables S3 and S4).

**Table 3:** A list of differentially expressed CGs (DECGs) identified through in silico expression analysis. For details of all the CGs see Tables S3 and S4.

No. of DECGs per MQTL	MQTLs with a specific number of expressed CGs, ranging from 1–8 (number of total MQTLs)
1	MQTL17, MQTL30, MQTL32, MQTL34, MQTL38, MQTL47, MQTL48, MQTL53, MQTL61 (9)
2	MQTL10, MQTL19, MQTL20, MQTL28, MQTL31, MQTL50, MQTL51 (7)
3	MQTL1, MQTL2, MQTL4, MQTL5, MQTL18, MQTL23, MQTL25, MQTL29, MQTL43, MQTL46, MQTL56, MQTL59, MQTL60 (13)
4	MQTL15, MQTL36, MQTL37 (3)
5	MQTL13, MQTL16, MQTL24, MQTL42, MQTL55, MQTL57 (6)
6	MQTL12, MQTL14, MQTL27 (3)
7	MQTL3, MQTL7 (2)
8	MQTL44 (1)

(*ii*) *GO analysis of CGs*. GO analysis of the above 385 CGs revealed a number of GO terms out of which some of the important and most abundant GO terms include those involved in biological processes like phosphorylation, protein ubiquitination, proteolysis, transmembrane transport, oxidation–reduction processes, etc. Similarly, important GO terms in molecular functions category included those involved in catalytic activity, ATP binding, protein binding, heme binding, iron ion binding, metal ion binding, transmembrane transporter activity, oxidoreductase activity, etc.

(*iii*) *Expression analysis of CGs*. Expression analysis of CGs allowed identification of DECGs, which encoded proteins belonging to the following classes: (i) R-domain containing proteins, (ii) transcription factors (Zn finger binding proteins, SANT/Myb domains, NAC domain, BTF3), (iii) transporters (mitochondrial carrier domains, sugar-phosphate transporter domain, sodium/carbon exchanger domain, SLC26A/SulP transporter), (iv) protein kinases, (v) proteins involved in calcium signaling, (vi) peptidases, (vii) proteins involved in oxidative stress (cytochrome P450) and (viii) proteins with domains like S1/P1 nuclease, six-bladed beta-propeller/strictosidine synthase, alpha-mannosyltransferase and peptidase T2 (Supplementary Table S4). A representative heat map of 29 important DECGs is shown in Fig. 4b; these CGs included the following: (i) 14 CGs encoding R domain containing proteins, (ii) 9 CGs known to be involved in disease resistance signaling pathways and (iii) 6 CGs having very high expression (FC ≥ 5 or ≤ − 5).

## Discussion

A new era of quantitative disease resistance involving use of DNA-based molecular markers for identification of QTLs for disease resistance started during early 1990s. The era started with the publication of the classical papers on interval mapping by Lander and Botstein^53,54^. Among plant systems (including wheat), interval mapping has been used to study the genetic control of all kinds of traits and resistance to stripe rust in wheat is no exception^55^. Thus, during the last 25 years, more than 70 reports involving mapping of > 350 QTLs (also described as quantitative resistance loci i.e., QRLs) for stripe rust appeared. A number of these QTLs also overlapped Yr genes that were already mapped.

It may be recalled that in the present study 61 MQTLs involving 184 QTLs were identified. This indicated roughly three-times reduction in the number of genomic regions controlling stripe rust resistance in wheat genome. Earlier, while conducting meta-QTL analysis in wheat for fusarium head blight, roughly five-fold reduction was involved^56^ and similar analysis showed nearly four-fold reduction in genomic regions controlling leaf rust resistance in wheat^35^. The absence of MQTLs on 5D, 6D, 7D agrees with earlier reports on QTL analysis^57–61^. Absence of QTLs on few chromosomes was nothing unusual, since in two earlier meta-QTL studies, no MQTLs for leaf rust resistance, were available on five chromosomes including 1D, 3D, 5A, 5D and 6D^35^, and no MQTLs for fusarium head blight were available on six chromosomes including 1D, 3D, 5D, 6D, 7B and 7D^56^. What is unusual is the absence of MQTLs on 5D and 6D in all the three studies (including the present study). A possible explanation for the limited QTLs located on the D sub-genome across various studies could be the low level of polymorphism associated with the D sub-genome. In the present study also, only 47 (13.3%) of the 353 QTLs used for metaQTL analysis belonged to the D sub-genome, suggesting that fewer QTLs are generally available on the D sub-genome.

Sixty-one MQTLs for stripe rust resistance in wheat (including four MQTLs derived from the QTLs belonging to durum wheat) is a fairly large number indicating occurrence of QTLs in close proximity, which agrees with a large number of Yr genes for stripe rust resistance reported in wheat genome. Occurrence of such a large number of genes/MQTLs should provide resistance against a large number of ever-evolving races of stripe rust, distributed in different wheat growing regions of the world^5^. Also, almost all the MQTLs (except MQTL36-4A) showed resistance against more than one pathotypes indicating that these MQTLs exhibit race non-specific resistance and may carry novel genes which may sometimes be involved in providing resistance against broad spectrum of pathotypes. Earlier, for resistance against fusarium head blight also, 65 MQTLs were identified. However, the number of MQTLs identified in this study far exceeds the number of MQTLs identified for leaf rust resistance (35)^35^. Perhaps this was due to relatively fewer QTL studies (19) available for meta-analysis in case of leaf rust resistance, so that in a recent report, the number of MQTLs for leaf rust resistance has risen to 75 MQTLs. In this recent study MQTLs are described as genetic map positions or gmQTLs (based on genetic map), since these were named on the basis of genetic map positions that were later converted into sequence mapped meta-QTLs or smQTL^36^. This new terminology for naming meta-QTLs as gmQTL and smQTL may be more widely used in future.

Most of the MQTLs identified in the present study controlled more than one traits (Table 2; Supplementary Table S2). This probably indicated either a tight linkage of genes for different traits, or occurrence of pleiotropic genes. This may also be attributed to a bias due to the use of related traits for identification of stripe rust resistance as also reported earlier in case of MQTL analysis for leaf rust resistance in wheat^35,36^.

It is also interesting to note that the positions of 24 MQTLs overlapped those for Yr genes; some of these Yr genes are already cloned and characterized (e.g., allelic Yr genes *Yr5* and *Yrsp* overlap MQTL17-2B and MQTL21-2B and *Yr15* overlap MQTL6-1B). The above three cloned Yr genes are known to encode proteins for NBS-LRR and therefore represent R genes^21,62^. These genomic regions carrying Yr genes and MQTL may be involved in controlling both the qualitative resistance (generally controlled by R genes) and quantitative resistance (controlled by QRLs) making them relatively more important.

We know that a large number of Yr genes have already been deployed for stripe rust resistance, but there is only one report available, where QTLs (*QYr.nafu-2BL* and *QYr.nafu-3BS*) were utilized for transfer of stripe rust resistance in wheat cultivars^63^. Some of the Yr genes which are still effective in India include *Yr5*, *Yr10, Yr15, Yrsp, Yr47, Yr57* and *Yr63*^64,65^. Three above cloned Yr genes (*Yr5*, *Yr15, Yrsp*) and four other Yr genes (*Yr10*, *Yr53, Yr61, Yr65, Yr69*) are also known to be effective worldwide^23,66^. Therefore, the MQTLs and the associated Yr genes may be utilized for developing a package to be used for improvement of stripe rust resistance.

It may also be recalled that there are 8 meta-QTLs, which occur in four pairs of MQTLs occurring in close proximity. These four pairs of MQTLs and five other MQTLs (8 + 5 = 13) listed in Table 4 may prove useful for breeding; therefore, we like to describe these MQTLs as breeders’ MQTLs. For selecting these breeders’ MQTLs, we utilized a number of criteria including the following two criteria suggested in an earlier study^38^. (i) The low CI and high average PVE of the MQTLs and (ii) the number of QTLs carried by individual MQTL. Additional criteria were also used in the present study for prioritizing and selecting breeders’ MQTLs. For instance, the relationship between MQTLs and the pathotypes occurring in specific wheat growing regions may be an important criterion. While doing this, we also have to keep in mind that virulence can also be quantitative in nature as mentioned earlier. MQTLs showing resistance against more than one pathotypes may also be an important criterion for achieving broad spectrum resistance. Such important MQTLs showing resistance against multiple pathogen races were also identified in a recent study on MQTL analysis conducted for tan spot resistance in wheat^37^.

**Table 4:** Some important MQTLs and their flanking markers, selected and described as breeders’ MQTLs [three MQTLs (MQTL20-2B, MQTL24-2D and MQTL18-2B) were classified into more than one categories and were therefore counted only once].

Important MQTLs (PVE%, stage of resistance, overlapped Yr genes)	Flanking markers	Important features
MQTL10-2A (45.00,APR)	wmc382—wpt-7024	Pathogen races ≥ 3; QTL ≥ 3 and traits ≥ 2
MQTL20-2B(40.26,APR + HTAP)	Excalibur_c5482_790—Excalibur_c26527_330	
MQTL24-2D(47.20,APR)	BS00093760_51—BS00066960_51	
MQTL40-4B(48.50,APR)	Tdurum_contig55414_154—Tdurum_contig56458_594	
MQTL18-2B (*Yr72*,APR + HTAP + ASR)	wpt-7026—kukri_c12117_134	PVE > 20%; number of pathogen races, initial QTL and traits, each ≥ 3 with co-located Yr genes
MQTL19-2B (APR + HTAP; *Yr43*,*Yr44*,*Yr53*,*Yrsp*,)	Bobwhite_c43708_93—Excalibur_c63327_110	
MQTL20-2B (APR + HTAP; *Yr43*, *Yr72*,)	Excalibur_c5482_790—Excalibur_c26527_330	
MQTL24-2D (APR; *Yr16*,)	BS00093760_51—BS00066960_51	
MQTL4-1B (APR + HTAP) and MQTL5-1B (APR + HTAP + ASR)	wsnp_JD_c19419_17536513—GENE-0129_123	Closely linked MQTL (MQTLs falling in same physical interval and cM ≤ 15 cM)
MQTL14-2B (APR) and MQTL15-2B (APR)	wpt-8460—GENE-0641_239	
MQTL17-2B (APR + HTAP) and MQTL18-2B (APR + HTAP + ASR)	BS00109853_51—kukri_c12117_134	
MQTL53-6B (APR + HTAP) and MQTL54-6B(APR + HTAP)	Tdurum_contig32579_121—Excalibur_c57840_227	

*PVE* phenotypic variance explained, *APR* adult plant resistance, *HTAP* high temperature adult plant resistance, *ASR* all stage resistance.

Further, almost all (60 out of 61) MQTLs contain original QTLs that are responsible for APR (or HTAP) and a solitary MQTL was based on ASR (including SR). This exclusive presence of QTLs for APR is perhaps due to QTL studies mostly conducted on APR.

The 385 CGs identified during the present study were subjected to a detailed study and were shown to encode a variety of proteins; at least some of these proteins are known to be involved in disease resistance (Table 3). Out of these 385 CGs, 265 CGs belonged to important classes of R genes which followed six out of the nine different mechanisms earlier proposed by Kourelis and van der Hoorn^67^ on the basis of the known protein products encoded by 314 cloned R genes in different crops. The six mechanisms followed by the R genes identified in the present study include direct and indirect perception of pathogen-derived molecules on the cell surface by receptor-like proteins and receptor like kinases, direct and indirect intracellular detection of pathogen-derived molecules by NLRs (NBS-LRR), detection through integrated domains and host reprogramming-mediated loss of susceptibility.

The differential expression of 144 CGs (for details, see Supplementary Table S4) at the seedling stage agrees with earlier reports^26,52,68^. These genes are largely involved in important processes like protein phosphorylation, photosynthesis, protein ubiquitination, transmembrane transport, oxidation–reduction processes, etc. which are relevant to disease resistance. For instance in an earlier study, a reduction in photosynthesis was shown to enhance stripe rust resistance due to the interaction of *Yr36*, encoding for *wheat kinase STARTI* (*WKSI*) with Psbo (a member of photosystem II)^69^ without having any adverse effect on yield. Similarly, in another study, several genes encoding PR (pathogenesis-related) proteins, involved in a number of defense responses were shown to get induced in response to stripe rust infection in a number of wheat lines carrying different genes for ASR (*YrTr1, Yr76, Yrsp, Yrexp2*) and HTAP (*Yr5, Yr59, Yr62* and *Yr7b*)^70^. A number of downstream genes, apparently similar to the CGs identified in the present study and involved in processes mentioned above were also identified in a transcriptome study conducted using a pair of introgression lines, which differed for *Yr5*^52^.

CGs underlying the MQTLs in wheat were also identified earlier for several traits including drought tolerance^34^, tan spot resistance^37^ and fusarium head blight resistance^56^. However, the criteria used by us was novel and not used in any of these earlier reports. For instance, in most of the earlier reports, the complete physical interval flanking the MQTL region was considered for identification of CGs. However, in the present study, we used 2 Mb region flanking the exact physical position of the MQTL based on the MQTL peak position available from the BioMercator software.

Fifty-nine (59) CGs out of the total 385 CGs belonged to MQTLs described as breeder’s MQTLs, and are, therefore, considered to be more important. Out of these 59 CGs, 32 CGs also showed differential expression and encoded important R proteins including S/TPK, SLC transporter, mitogen-activated protein (MAP) kinase, UDP-glucosyltransferases, S1/P1 nuclease, etc. The known roles of some of the important CGs (shown in Fig. 4) during disease resistance can be summarised as follows: (i) *STPK-V*, a member of *Pm1* gene was reported to confer powdery mildew resistance in wheat^71^. (ii) NBS-LRR domain containing genes are the protein products of the cloned Yr genes like *Yr10, Yr5*, etc. as mentioned earlier^21,62^. (iii) *TaMAPK4*, a type of MAPK gene is reported to act as a positive regulator of stripe rust resistance in wheat^72^. (iv) The above transporters may also possibly represent Yr genes similar to *Yr46* which was shown to encode a hexose transporter^19^. (v) UDP-glucosyltransferases were earlier reported to show differential expression due to stripe rust infection in wheat genotypes indicating their role in *Yr39* mediated stripe rust resistance^73^. Some other important CGs like those encoding for WRKY domains, ankyrin repeat and F-box domain containing genes were also identified in different MQTLs, although the expression data was not available for these genes. WRKY and ankyrin repeat domain containing genes were recently found to encode for proteins of cloned *YrU* gene^22^. Similarly, a F-box domain containing gene was identified as a CG underlying *YrR39* that has been used for improvement of stripe rust resistance^74^.

In summary, the present study allowed us to identify 5 MQTLs and 4 pairs of closely linked MQTLs that may be used by breeders for developing high yielding stripe rust resistant wheat cultivars (Table 4). Eight of these 13 genomic regions overlapped the known Yr genes. Some of the important CGs like those encoding for NBS-LRR proteins, WRKY proteins, transporters, UDP-glucosyltransferases and MAP kinases may either correspond for important known Yr genes or involved in downstream signalling processes during wheat- *Puccinia striiformis* interaction. Therefore, these CGs may be used for molecular breeding after validation. These MQTLs may be used for MAS in wheat breeding after necessary validation and may also be used for fine-mapping leading to cloning. Some of these genes may, however, function through their effect on other genes that are directly involved in stripe rust resistance. For instance, overexpression of *TaWRKY62* provided seedling resistance to stripe rust by activating a variety of genes including PR protein genes, salicylic/jasmonic acid responsive genes and ROS associated genes^75^. In another study, induced overexpression of *TaLHY* (a type of pf MYB TF) in leaf blade and sheath reduced the damage caused by stripe rust^76^. This knowledge may prove useful for validating some CGs identified in the present study.

Gene editing and induced mutations are two other approaches, which are still unexplored in case of stripe rust resistance except a single study, where the function of *Yr15* gene (encoding *wheat tandem kinase 1* or *WKS1*) was validated throught induced mutations^20^. In this study, using a resistant line, EMS induced mutations in *Yr15* were successfully obtained for susceptibility. The resulting susceptible lines carried mutant allele leading to changes in three amino acids (Gly54, Ala149 and Ala460) causing disruption in gene function, thus validating the role of *WSK1* in resistance. A similar strategy may be used for at least three cloned genes (*Yr5/Yrsp, Yr7* and *Yr10*) which overlap the marker interval carrying two important MQTLs and the associated CGs (Table 4; Fig. 4a,b). Techniques involving gene editing or base editing may also be used for the above cloned Yr genes and the CGs after identification of causal SNPs involved in providing stripe rust resistance through CGAM approach. CRISPR/Cas9 has already been used for editing three genes (*TaABCC6, TaNFXL1*, and *TansLTP9*) for resistance against fusarium head blight^77^, thus demonstrating the possibility of using gene/base editing. The present study thus provided resources that may be used in future for wheat breeding and for basic studies involving stripe rust resistance.

## Materials and methods

### Search for QTLs and input file preparation

The information on QTLs for stripe rust resistance reported during the past 20 years (2000 to 2020) was obtained from WheatQTLdb^28^ (Supplementary Table S1) and other published literature. The detailed information from each study was collected on the following aspects: (i) types of mapping populations and their parents, (ii) size of mapping population, (iii) pathotype(s) used for phenotyping, (iv) methods of QTL mapping, (v) position of QTLs and markers flanking the QTLs, (vi) logarithm of odds (LOD) value, and (vii) R^2^ values of the QTLs. Only QTLs with complete information required for meta-analysis were retained for final analysis. The two input data text files used for meta-QTL analysis included the genetic map file and QTL information file from each study following the instructions provided in the BioMercator v3/v4 manual^40^.

### Construction of consensus genetic map

A consensus genetic map involving SSR, DArT and SNP markers was constructed using LPmerge software^41^. For this purpose, five individual genetic linkage maps each from different studies were used as reference maps^42–46^. Markers flanking individual QTL regions were also included for construction of a consensus genetic map.

### Projection of QTLs on consensus map

The original QTLs were projected onto the consensus map using the QTL projection tools (QTL Proj) available in BioMercator v4.2. The QTLs which could not be projected onto the consensus map were excluded. For the projection of QTLs, a scaling rule between the marker interval of the original QTL and the corresponding interval on the consensus map was used^47^. The new confidence interval (CI) for MQTLs on the consensus linkage group was computed using Gaussian distribution^47^.

### Meta-analysis of QTLs and identification of MQTLs

MQTL analysis was conducted using BioMercator v4.2^40,48^. Following two different approaches were used based on the number of QTLs on each individual chromosome for conducting MQTL analysis: (i) If the number of QTLs on an individual chromosome was ≤ 10, the approach suggested by Goffinet and Gerber^49^ was used; and (ii) if the number of initial QTLs on an individual chromosome was > 10, the approach suggested by Veyrieras et al.^47^ was used. For identification of the MQTLs, QTL model having the lowest Akaike information criterion (AIC) value was considered (AIC value estimates the relative amount of data lost by different statistical models). Quality of the model depended on the assumption that lesser the information loss, higher will be the quality of that model^50^. Whenever, more than one MQTLs were located in the same physical interval (genetic/confidence interval < 15 cM), these were treated as closely linked MQTLs.

### Yr genes associated with MQTL

The sequences of markers associated with Yr genes were blasted against the wheat reference genome version 49 (Ensembl_Release 49) available in the Ensembl Plants database. This allowed identification of physical coordinates of the associated markers. These physical intervals containing the Yr genes were compared with the physical coordinates of the MQTL regions to identify association of Yr genes with individual MQTLs.

### Identification of CGs underlying the MQTLs

The CGs underlying the MQTLs were identified in the 1 Mb interval on either side of the peak position of the MQTL (total 2 Mb interval). For this purpose, the options available in biomart tool of Ensembl Plants were utilized. The following steps were used to identify CGs: (i) the physical coordinates of the MQTLs were extracted by BLAST search of the sequences of markers (retrieved from GrainGenes, CerealDB or JBrowse) flanking the MQTLs in the wheat reference genome sequence (*Triticum_aestivum* Ensembl_Release 49) available in Ensembl Plants database. (ii) The physical interval (in Mb) for an individual MQTL was calculated using the genetic confidence interval (in cM) of the MQTL regions. For this purpose, the physical interval (in Mb, calculated from the coordinate information of the MQTL) was divided by the genetic interval (in cM) and the distance in units of bases per cM was calculated. (iii) Actual physical position of the MQTL was calculated and 1 Mb region on either side of the MQTL peak (total 2 Mb interval) was used for identification of the putative CGs associated with the respective MQTL region. (iv) Annotations of CGs was undertaken on the basis of the domain in the corresponding protein sequences, which were obtained using InterPro database.

### Gene ontology (GO) and in silico expression analysis of CGs

GO analysis was conducted using Biomart tool available in Ensemble Plants. In silico expression of CGs was conducted using expression data available in expVIP database^51^. The available expression data used in the present study belonged to two different studies. In the first study, Zhang et al.^26^ inoculated seven-day old seedlings of wheat variety N9134 using race CYR31 for yellow rust and race E09 for powdery mildew. The inoculated leaves were harvested at 0, 1, 2, and 3 dpi and expression data were collected for both yellow rust and powdery mildew (using 0 dpi as control). In the second study, Dobon et al.^52^ used two different genotypes, namely Vuka (susceptible) and Avocet-*Yr5* (resistant), which were inoculated with Pst isolate 87/66 at three leaf stage. Leaf samples were collected at 0, 1, 2, 3, 5, 7, 9, and 11 days post-inoculation (dpi) in susceptible genotype Vuka, but for only five days (at 0, 1, 2, 3, and 5 dpi) for the resistant line Avocet-*Yr5*. In this second study, expression data was collected for genes in both the host and the pathogen.

The data on expression in expVIP was available as log_2_ transformed tpm (transcripts per million) values. Only genes (CGs) showing FC ≥ 2 (upregulation, twofold or more) or FC ≤ − 2 (downregulation, twofold or more) were accepted as showing differential expression in the form of fold changes estimated by comparing tpm values under stress vs. control. The results of such differentially expressed CGs were depicted in the form of heatmaps generated using online tool Morpheus that is available at https://software.broadinstitute.org/morpheus/.

## Supplementary Information

Supplementary Information.
